# Neurometabolic and Neuroinflammatory Consequences of Obesity: Insights into Brain Vulnerability and Imaging-Based Biomarkers

**DOI:** 10.3390/ijms27020958

**Published:** 2026-01-18

**Authors:** Miloš Vuković, Igor Nosek, Milica Medić Stojanoska, Duško Kozić

**Affiliations:** Faculty of Medicine, University in Novi Sad, 21000 Novi Sad, Serbia; igor.nosek@mf.uns.ac.rs (I.N.); milica.medicstojanoska@kcv.rs (M.M.S.); dusko.kozic@mf.uns.ac.rs (D.K.)

**Keywords:** obesity, brain vulnerability, neuroinflammation, neurometabolism, magnetic resonance spectroscopy, multimodal neuroimaging, insulin resistance, cognitive impairment

## Abstract

Obesity is a systemic metabolic disorder characterized by chronic low-grade inflammation and insulin resistance, with growing evidence indicating that the brain represents a primary and particularly vulnerable target organ. Beyond peripheral metabolic consequences, obesity induces region-specific structural, functional, and biochemical alterations within the central nervous system, contributing to cognitive impairment, dysregulated energy homeostasis, and increased susceptibility to neurodegenerative diseases. This narrative review examines key neurometabolic and neuroinflammatory mechanisms underlying obesity-related brain vulnerability, including downstream neuroinflammation, impaired insulin signaling, mitochondrial dysfunction, oxidative stress, blood–brain barrier disruption, and impaired brain clearance mechanisms. These processes preferentially affect frontal and limbic networks involved in executive control, reward processing, salience detection, and appetite regulation. Advanced neuroimaging has substantially refined our understanding of these mechanisms. Magnetic resonance spectroscopy provides unique in vivo insight into early neurometabolic alterations that may precede irreversible structural damage and is complemented by diffusion imaging, volumetric MRI, functional MRI, cerebral perfusion imaging, and positron emission tomography. Together, these complementary modalities reveal microstructural, network-level, structural, hemodynamic, and molecular alterations associated with obesity-related brain vulnerability and support the concept that such brain dysfunction is dynamic and potentially modifiable. Integrating neurometabolic and multimodal neuroimaging biomarkers with metabolic and clinical profiling may improve early risk stratification and guide preventive and therapeutic strategies aimed at preserving long-term brain health in obesity.

## 1. Introduction

**Figure 1:**
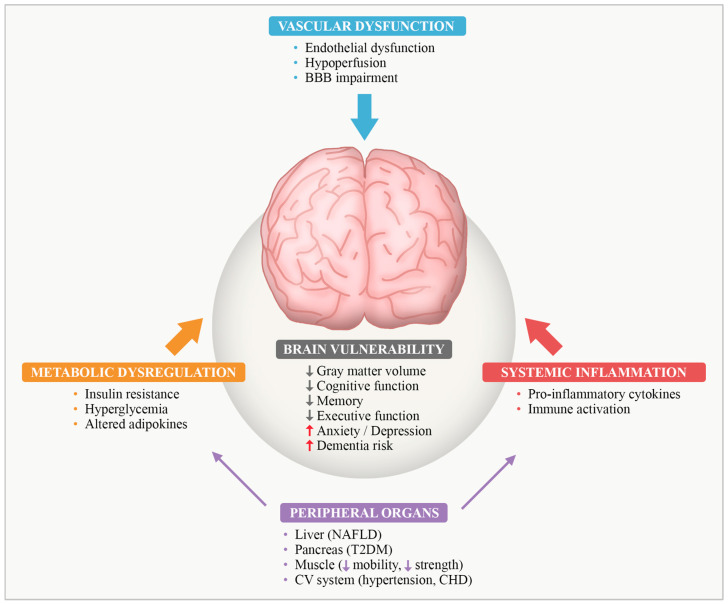
Systemic pathways linking obesity to brain vulnerability. Integrative schematic illustrating how obesity-related metabolic dysregulation, systemic low-grade inflammation, and vascular dysfunction converge to impair periphery–brain communication. Metabolic disturbances, including insulin resistance, hyperglycemia, and altered adipokine signaling, together with chronic immune activation and elevated pro-inflammatory cytokines, and vascular alterations such as endothelial dysfunction, cerebral hypoperfusion, and blood–brain barrier (BBB) impairment, synergistically promote structural, cognitive, and affective brain alterations. Peripheral organ dysfunction further amplifies systemic metabolic and inflammatory stress, increasing the risk of cognitive decline and dementia. Abbreviations: BBB, blood–brain barrier; CHD, coronary heart disease; CNS, central nervous system; CV, cardiovascular; NAFLD, non-alcoholic fatty liver disease; T2DM, type 2 diabetes mellitus. This figure represents an original schematic illustration created by the authors, providing a conceptual synthesis informed by the literature.

Obesity exerts widespread effects on human health through complex metabolic and inflammatory mechanisms that affect multiple organ systems, including the central nervous system [1]. Beyond its systemic consequences, obesity is increasingly recognized as a condition that profoundly alters brain structure and function, establishing the brain as a vulnerable target organ in the pathogenesis of obesity-related complications [2,3,4]. Recent evidence highlights that obesity-associated low-grade inflammation disrupts key periphery–brain communication pathways, particularly by impairing blood–brain barrier (BBB) integrity and promoting maladaptive neuroimmune signaling across central–peripheral interfaces [5,6]. Beyond hypothalamic circuits, systemic metabolic inflammation can induce region-specific neuroinflammatory responses through cytokine-mediated signaling and vascular–immune interactions [7]. Figure 1 schematically illustrates the integrative metabolic, inflammatory, and vascular pathways through which obesity exerts multisystem effects and promotes brain vulnerability.

Importantly, the relationship between obesity and brain dysfunction is bidirectional. While altered central regulation of appetite, reward processing, and executive control may contribute to weight gain, obesity itself represents a major upstream driver of neurological vulnerability. Chronic low-grade systemic inflammation, metabolic dysregulation, hormonal imbalance, and vascular dysfunction associated with obesity promote BBB impairment, microglial activation, and sustained neuroinflammatory signaling [8,9,10]. These mechanisms contribute to structural, metabolic, and functional brain alterations, thereby positioning obesity primarily as an upstream driver of neurological vulnerability while acknowledging the bidirectional interactions between central dysregulation and metabolic disease.

## 2. Neuroinflammation as a Central Mechanism

Chronic low-grade systemic inflammation represents a hallmark of obesity and a key mechanism linking excess adiposity to brain dysfunction [4,11]. Unlike acute inflammatory responses, obesity-related inflammation is persistent and low in intensity, yet sufficient to disrupt metabolic homeostasis and neural function. The hypothalamus, a central regulator of energy balance, is particularly susceptible to inflammatory insults induced by high-fat diets [12].

Experimental and clinical studies indicate that hypothalamic inflammation may develop early during exposure to high-calorie diets, even before overt weight gain becomes apparent [13]. Activation of microglia within the hypothalamus leads to increased production of pro-inflammatory cytokines, including tumor necrosis factor alpha (TNF-α), interleukin-1 beta (IL-1β), and interleukin-6 (IL-6), which interfere with leptin and insulin signaling and impair central regulation of energy homeostasis [14,15,16].

These processes contribute to the development and amplification of central insulin and leptin resistance, which may represent both predisposing factors for obesity and downstream consequences of chronic metabolic dysregulation [17,18,19,20]. Importantly, emerging evidence suggests that obesity-related neuroinflammatory changes are not confined to hypothalamic circuits but involve broader periphery–brain communication pathways. Systemic metabolic inflammation, vascular dysfunction, and blood–brain barrier alterations facilitate neuroimmune signaling and promote region-specific neuroinflammatory responses [21].

**Figure 2:**
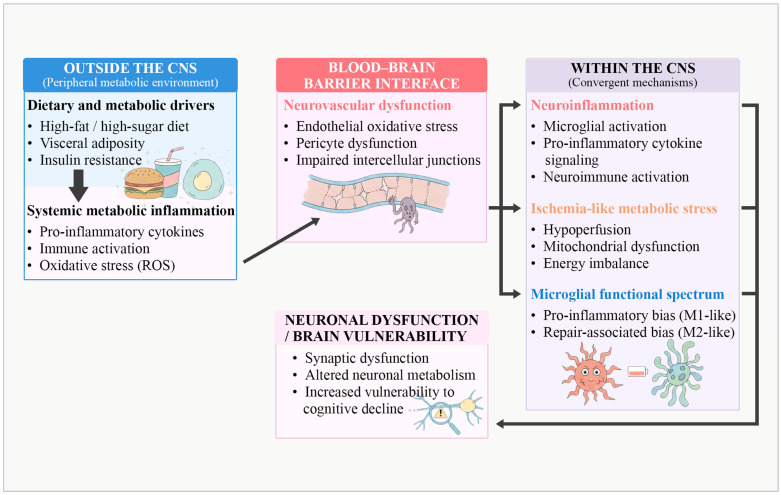
Integrative mechanisms linking systemic metabolic inflammation to neuroinflammatory and neurovascular brain vulnerability in obesity. Schematic representation of the periphery–brain pathways through which obesity-related metabolic and inflammatory disturbances promote central nervous system vulnerability. Peripheral dietary and metabolic drivers induce systemic metabolic inflammation characterized by immune activation, oxidative stress, and elevated pro-inflammatory cytokines. These signals converge at the blood–brain barrier interface, leading to neurovascular dysfunction, including endothelial oxidative stress, pericyte impairment, and disruption of intercellular junctions. Within the central nervous system, convergent mechanisms involving microglial activation, cytokine-mediated neuroinflammation, ischemia-like metabolic stress, and altered microglial functional states contribute to synaptic dysfunction, altered neuronal metabolism, and increased susceptibility to cognitive decline. Abbreviations: CNS, central nervous system; ROS, reactive oxygen species. This figure represents an original schematic illustration created by the authors, providing a conceptual synthesis informed by the literature.

The integrative mechanisms linking obesity-related systemic metabolic inflammation to neuroinflammatory and neurovascular brain vulnerability are illustrated in Figure 2. Microglial activation in obesity spans a functional spectrum, ranging from pro-inflammatory, M1-like phenotypes to repair-associated, M2-like phenotypes. In parallel, obesity-associated vascular dysfunction and cerebral hypoperfusion may induce ischemia-like metabolic stress, further amplifying neuroinflammatory signaling and contributing to neuronal dysfunction [21,22].

Recent study emphasizes that obesity-related neuroinflammation extends beyond the hypothalamus and involves widespread cortical and subcortical regions through immune–metabolic signaling pathways [21]. The BBB, particularly specialized hypothalamic interfaces involving tanycytes, plays a crucial role in regulating the entry of peripheral metabolic and inflammatory signals into the brain. Hypothalamic regions exhibit barrier plasticity and are anatomically predisposed to peripheral signal exposure, enabling dynamic communication with key hypothalamic nuclei involved in metabolic regulation [23,24,25]. Increased permeability facilitates the entry of inflammatory factors into the brain parenchyma, amplifying neuroinflammatory responses and neuronal stress.

Both microglia and astrocytes actively participate in obesity-induced neuroinflammation. Activated microglia initiate and propagate inflammatory signaling cascades, while astrocytes further amplify these responses through the release of pro-inflammatory mediators [26,27,28,29,30]. In obesity, metabolic disturbances, including elevated dietary saturated fatty acids, hyperglycemia, and oxidative stress, act as upstream triggers of innate immune signaling within the central nervous system.

**Figure 3:**
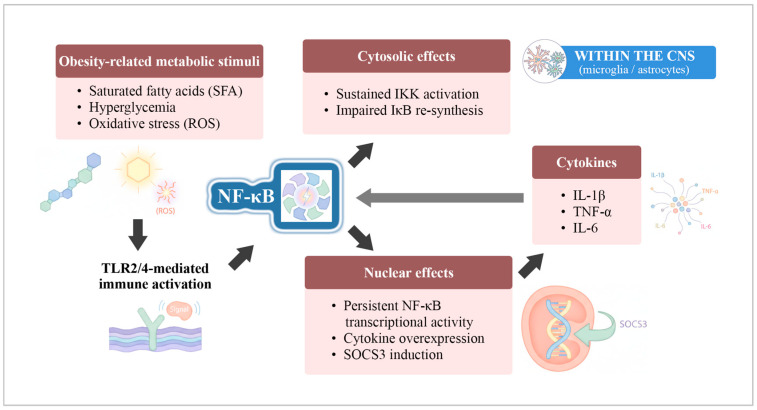
Chronic metabolic activation of NF-κB signaling in obesity-related neuroinflammation. Schematic representation of sustained, metabolically driven NF-κB activation in glial cells within the central nervous system. Obesity-related metabolic stimuli engage Toll-like receptor 2/4 signaling, resulting in persistent NF-κB activity characterized by prolonged IκB kinase (IKK) activation, impaired IκB re-synthesis, and continuous nuclear transcriptional signaling. The diagram highlights cytokine overexpression, SOCS3 induction, and a self-amplifying neuroinflammatory feedback loop, illustrating a chronic, obesity-specific inflammatory state. Abbreviations: TLR2/4, Toll-like receptor 2/4; NF-κB, nuclear factor kappa B; IKK, IκB kinase; IκB, inhibitor of κB; SFA, saturated fatty acids; IL-1β, interleukin-1 beta; TNF-α, tumor necrosis factor alpha; IL-6, interleukin-6; SOCS3, suppressor of cytokine signaling 3; CNS, central nervous system; ROS, reactive oxygen species. This figure represents an original schematic illustration created by the authors, providing a conceptual synthesis informed by the literature.

These metabolic stimuli activate Toll-like receptor 2/4 (TLR2/4)-mediated pathways in glial cells, leading to sustained activation of nuclear factor kappa B (NF-κB), a central regulator of inflammatory gene transcription. Chronic NF-κB signaling promotes persistent nuclear transcriptional activity, resulting in overexpression of pro-inflammatory cytokines and induction of suppressor of cytokine signaling 3 (SOCS3), thereby driving neuroinflammatory amplification through a self-reinforcing metabolic–immune loop [31,32,33,34]. The molecular signaling cascade linking obesity-related metabolic stimuli to chronic neuroinflammatory activation is schematically illustrated in Figure 3.

## 3. Neuroendocrine Regulation of Neuroinflammation in Obesity

Beyond metabolic and immune mechanisms, obesity-related neuroinflammation is modulated by neuroendocrine pathways. Dysregulation of the hypothalamic–pituitary–adrenal (HPA) axis and altered glucocorticoid metabolism have been implicated in obesity and metabolic disease, particularly through enhanced local cortisol regeneration mediated by 11β-hydroxysteroid dehydrogenase type 1 (11β-HSD1) [35]. These neuroendocrine alterations may influence neuroinflammatory signaling and stress-responsive brain circuits. In parallel, the brain is not merely a passive target of peripheral hormones but is capable of autonomous steroid metabolism, including the local synthesis and interconversion of neuroactive steroids such as dehydroepiandrosterone (DHEA) and dehydroepiandrosterone sulfate (DHEAS), which modulate inflammatory and neuroprotective processes within the central nervous system [36].

Glucocorticoid (type 1) and mineralocorticoid (type 2) receptors are widely expressed in the hippocampus, prefrontal cortex, and limbic regions, where they play a central role in regulating stress responsivity, synaptic plasticity, neurogenesis, and inflammatory signaling [37]. Alterations in glucocorticoid signaling, including changes in receptor density and sensitivity, have been documented with aging and chronic stress exposure, contributing to increased vulnerability to neuroinflammatory processes and neurodegenerative conditions such as Alzheimer’s disease [37]. In the context of obesity and metabolic disease, dysregulated glucocorticoid availability, partly mediated by enhanced local cortisol regeneration via 11β-HSD1, may further exacerbate these stress-related and inflammatory pathways [35].

Obesity-related neuroinflammation extends beyond resident microglial activation and involves complex interactions with circulating and infiltrating immune cells [21]. Chronic low-grade systemic inflammation associated with obesity is linked to alterations in BBB function, which may facilitate enhanced peripheral immune-to-brain signaling and contribute to central neuroinflammatory responses [21,38]. Monocytes and macrophages represent key mediators of this process, as obesity favors a shift toward pro-inflammatory phenotypes that amplify cytokine production and sustain neuroinflammatory signaling within vulnerable brain regions [21].

In addition to monocyte-derived cells, adaptive immune components contribute to obesity-associated inflammatory dysregulation [21,39]. Altered T-lymphocyte profiles, including a shift toward pro-inflammatory subsets and a relative reduction in regulatory T-cell populations, have been implicated in sustaining chronic inflammatory responses in obesity [39]. Moreover, antigen-presenting cells, including dendritic cells and macrophages, may further contribute to immune signaling by enhancing antigen presentation and shaping cytokine-dependent T-cell polarization, thereby influencing inflammatory amplification and its regulation [39].

Importantly, peripheral immune cells may exert dual roles within the brain, contributing not only to inflammatory injury but also to tissue repair and immune regulation, depending on their activation state and the local microenvironment [21,39]. Dysregulation of this finely balanced immune response in obesity may favor persistent neuroinflammation, oxidative stress, and synaptic dysfunction, thereby strengthening the association between systemic metabolic disturbances and central nervous system vulnerability [21,38,39].

Sex-related differences represent an important but often underappreciated modifier of neuroinflammatory processes relevant to obesity-related brain vulnerability. Experimental and clinical evidence indicates that males and females exhibit distinct neuroimmune responses, driven largely by the modulatory effects of sex hormones on microglial activation, cytokine production, and cellular metabolic signaling pathways [40,41]. Estrogens exert predominantly anti-inflammatory and neuroprotective effects by attenuating microglial reactivity and supporting neuronal and mitochondrial function, whereas androgens and progesterone display context-dependent immunomodulatory actions within the central nervous system [40].

In females, the decline in estrogen levels during menopause is associated with increased central inflammation, oxidative stress, and heightened vulnerability to metabolic and neurodegenerative disorders [41]. Obesity may further exacerbate these processes by amplifying hormonal imbalance and systemic inflammation, potentially contributing to sex-specific differences in cognitive performance and brain vulnerability [41,42]. These observations underscore the importance of considering biological sex and hormonal status when interpreting neuroinflammatory mechanisms and neuroimaging findings in obesity.

## 4. Insulin Resistance and Neurodegenerative Pathways

Insulin resistance represents a central biological link between obesity, metabolic syndrome, and brain vulnerability; however, it should not be viewed exclusively as a downstream consequence of excess adiposity. Genetic predisposition, receptor-level alterations, and impaired intracellular insulin signaling may precede weight gain and can be further aggravated by obesity-related inflammation and lipid overload, thereby amplifying metabolic and neurocognitive risk [17,18]. Within the central nervous system, insulin resistance disrupts neuronal glucose utilization, synaptic plasticity, and neurotrophic signaling, thereby increasing susceptibility to neurodegenerative processes and cognitive dysfunction [43].

Similarly, leptin resistance exhibits a bidirectional relationship with obesity. While chronic hyperleptinemia associated with increased adipose tissue mass promotes receptor desensitization, hypothalamic leptin resistance may also arise from genetic variability, impaired leptin transport across the blood–brain barrier, or saturation of leptin receptors within key appetite-regulating nuclei [19,20]. Under physiological conditions, leptin acts as a critical protective signal limiting food intake and promoting energy homeostasis. Disruption of this signaling axis therefore contributes to both the development and persistence of obesity and may have downstream consequences for brain function and metabolic regulation.

Insulin plays an essential role in brain function, particularly in regions involved in learning and memory, such as the hippocampus [44,45]. In obesity, progressive peripheral insulin resistance leads to impaired insulin transport across the BBB and the development of central insulin resistance [46,47]. This condition is associated with reduced cerebral glucose utilization, diminished neuronal activity, and impaired cognitive performance [47,48].

Structural imaging studies have linked insulin resistance to reductions in brain volume and poorer performance on memory and visuospatial tasks, especially in middle-aged and older individuals [49,50]. Aging and type 2 diabetes mellitus (T2DM) further exacerbate these effects by reducing insulin availability in the brain and impairing insulin receptor signaling at the neuronal level [51,52].

Mitochondrial dysfunction and oxidative stress represent tightly interconnected and mutually reinforcing mechanisms linking insulin resistance to neurodegeneration. Impaired mitochondrial oxidative phosphorylation promotes excessive production of reactive oxygen species (ROS), while sustained oxidative stress further damages mitochondrial DNA (mtDNA), proteins, and membranes, thereby exacerbating mitochondrial dysfunction [53,54]. Rather than a unidirectional process, this vicious cycle amplifies neuronal vulnerability under conditions of chronic metabolic stress.

In obesity and insulin-resistant states, inflammatory signaling further accelerates mitochondrial dysfunction and energetic failure [54]. Activated microglia and infiltrating immune cells generate reactive oxygen and nitrogen species, intensifying oxidative damage and disrupting neuronal energy metabolism [55]. These processes impair synaptic function, calcium homeostasis, and axonal transport, thereby facilitating neurodegenerative cascades [54,55].

Cellular resilience to oxidative stress critically depends on the availability of nicotinamide adenine dinucleotide (NAD^+^) and its phosphorylated form NADPH. NAD^+^ depletion compromises mitochondrial bioenergetics and sirtuin-mediated protective pathways, whereas reduced NADPH availability limits antioxidant defenses, including glutathione regeneration and redox buffering. In the context of metabolic dysfunction, altered NAD^+^/NADH and NADP^+^/NADPH ratios may therefore represent key mediators linking insulin resistance, inflammation, and progressive neuronal injury [53,56].

## 5. Structural, Functional, and Cognitive Consequences

Neuroimaging studies employing magnetic resonance imaging (MRI) have consistently demonstrated obesity-related alterations in brain structure and function, particularly involving hypothalamic injury [26,57]. Reduced gray matter volume has been observed in the prefrontal cortex, hippocampus, and temporal regions, changes that are associated with impaired executive function, decision-making, and impulse control [58,59]. Importantly, recent pediatric neuroimaging study indicates that differences in hippocampal volume and memory performance can already be detected in childhood among individuals at increased familial risk for obesity, even in the absence of overt overweight or obesity, suggesting that obesity-related brain vulnerability may emerge early in life [60]. Disruption of BBB integrity further exacerbates neuroinflammation and neuronal vulnerability [24,61].

Functional alterations include dysregulation of dopaminergic reward pathways, particularly reduced D2 receptor activity in the nucleus accumbens, which may promote compensatory overeating and preference for energy-dense foods [62]. Magnetic resonance spectroscopy (MRS) studies complement these findings by demonstrating reductions in N-acetylaspartate (NAA), a marker of neuronal integrity and metabolic efficiency, suggesting impaired cerebral energy metabolism in obesity [63,64].

Clinically, obesity-related neurobiological alterations manifest as cognitive deficits involving attention, memory, and executive function, along with an increased prevalence of mood disorders and sleep disturbances [65,66]. These outcomes arise from the cumulative effects of chronic neuroinflammation, central insulin resistance, vascular dysfunction, and impaired cerebral perfusion, which together increase vulnerability to neurodegenerative processes, particularly Alzheimer’s disease [67,68,69]. While dopaminergic alterations have been described in obesity, current evidence does not support a consistent association with an increased risk of Parkinson’s disease [70,71].

**Figure 4:**
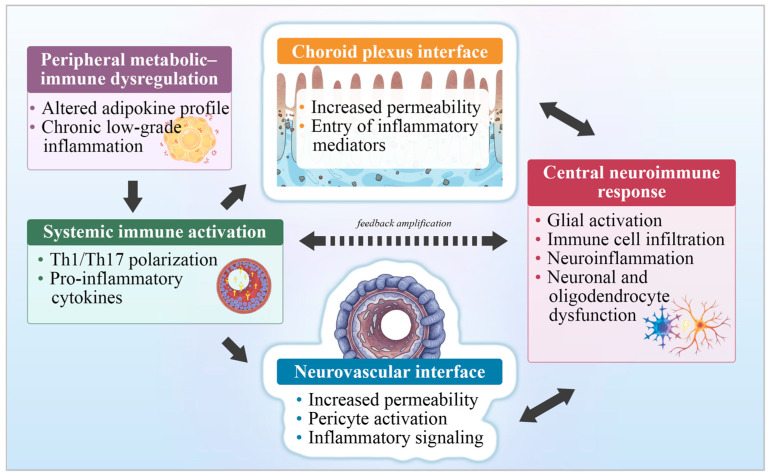
Neuroimmune interfaces mediating obesity-related brain vulnerability. Schematic overview of the neuroimmune interfaces through which obesity-related peripheral immune disturbances contribute to central nervous system vulnerability. Peripheral metabolic–immune dysregulation promotes systemic immune activation, which signals to the brain primarily via the neurovascular and choroid plexus interfaces. These interfaces mediate bidirectional communication with the central neuroimmune response, facilitating glial activation, immune cell infiltration, and neuroinflammatory processes that contribute to neuronal and oligodendrocyte dysfunction. A dashed bidirectional loop between systemic immune activation and the central neuroimmune response indicates feedback amplification of inflammatory signaling. Abbreviations: CNS, central nervous system; Th1, T helper 1 cells; Th17, T helper 17 cells. This figure represents an original schematic illustration created by the authors, providing a conceptual synthesis informed by the literature.

Figure 4 provides an integrative schematic of the neuroimmune interfaces through which obesity-related peripheral immune disturbances are transmitted to the central nervous system, highlighting the bidirectional interactions between systemic immune activation and central neuroimmune responses that underlie brain vulnerability.

## 6. Implications for Prevention and Intervention

Interventions such as caloric restriction have been shown to benefit brain aging and slow progression of neurodegenerative processes, possibly via metabolic and neuroprotective mechanisms, highlighting the potential of metabolic regulation strategies for long-term brain health [72].

Emerging evidence suggests that weight loss and metabolic interventions may partially reverse obesity-related alterations in brain metabolic signaling, particularly within hypothalamic circuits, supporting the concept of dynamic and potentially modifiable brain vulnerability [16].

Recent longitudinal neuroimaging study further supports this concept by demonstrating that weight loss–associated improvements in metabolic and low-grade inflammatory markers are accompanied by favorable shifts in brain aging trajectories, as reflected by reductions in brain-predicted age difference, and are paralleled by improvements in specific domains of cognitive performance [73].

Importantly, several obesity-related inflammatory pathways discussed above have direct or indirect neurometabolic correlates detectable by magnetic resonance spectroscopy (MRS). Pro-inflammatory cytokines and innate immune signaling, including TNF-α, IL-6, and TLR2/4 activation, are associated with neuronal metabolic stress, altered membrane turnover, and glial activation. These processes may manifest on MRS as reductions in NAA, reflecting neuronal dysfunction, and increases in choline-containing compounds (Cho) and myo-inositol (mI), which are commonly interpreted as markers of membrane remodeling and glial reactivity. Thus, MRS provides a non-invasive window into the downstream neurometabolic consequences of obesity-related neuroinflammation.

## 7. Magnetic Resonance Spectroscopy as a Tool for Assessing Obesity-Related Brain Vulnerability

MRS represents a non-invasive neuroimaging technique that enables in vivo assessment of brain metabolism by quantifying specific neurometabolites associated with neuronal integrity, membrane turnover, glial activity, and energy metabolism [74]. Unlike conventional MRI, which primarily provides structural information, MRS offers biochemical insights into pathophysiological processes that may precede overt structural brain changes. Recent systematic review has consolidated the role of MRS in identifying neurometabolic alterations in obesity and related metabolic disturbances. The most consistently reported changes include reduced NAA and increased mI across multiple brain regions, which may reflect obesity-related metabolic dysregulation and neuroinflammatory signaling and contribute to altered cerebral biochemistry [75].

Proton MRS (^1^H-MRS) is the most widely used spectroscopic approach in clinical and research settings due to its high sensitivity and compatibility with standard MRI systems [74]. By analyzing metabolite concentrations or metabolite ratios within selected brain regions, MRS allows indirect evaluation of neuronal and glial function under physiological and pathological conditions. In the context of obesity, this technique is particularly valuable, as metabolic and inflammatory brain alterations may occur before irreversible neuronal damage becomes apparent.

Among the neurometabolites detectable by MRS, NAA is considered a marker of neuronal integrity and mitochondrial function. NAA is synthesized in neuronal mitochondria and reflects both neuronal viability and metabolic efficiency [76]. Reduced NAA levels have been consistently reported in conditions associated with neuronal dysfunction or energy failure, including ischemia, neurodegeneration, and inflammatory brain disorders [77]. In obese individuals, decreases in NAA, particularly within frontal and subcortical regions, suggest impaired neuronal metabolism and increased vulnerability to metabolic stress [78,79]. Importantly, reductions in NAA do not necessarily indicate irreversible neuronal loss, as reversible decreases have been documented in several neurological conditions following metabolic or inflammatory stabilization [80,81,82].

Choline-containing compounds (Cho) reflect membrane phospholipid turnover and cellular membrane remodeling. Elevated Cho levels are commonly observed in disorders characterized by increased membrane synthesis or breakdown, including neuroinflammation, demyelination, and gliosis [83,84,85,86,87]. In obesity, increased Cho concentrations are thought to reflect chronic low-grade neuroinflammation and glial activation, consistent with evidence of inflammatory signaling within the central nervous system [14,15]. Regional variability in Cho levels further suggests differential susceptibility of brain regions to obesity-related inflammatory stress [74].

Creatine (Cr) plays a central role in cellular energy metabolism and is often used as an internal reference in MRS analyses due to its relative stability across many physiological conditions [88,89]. Although absolute Cr concentrations are typically preserved in obesity, altered ratios involving Cr may indicate subtle disturbances in cerebral energy homeostasis, particularly in the setting of insulin resistance and mitochondrial dysfunction [90,91].

**Figure 5:**
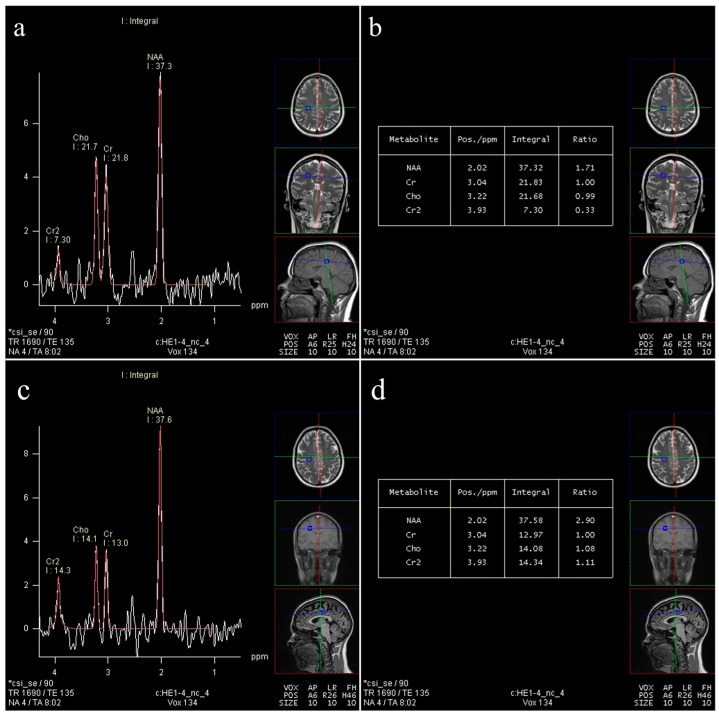
Representative multivoxel MRS spectra in obesity and healthy control [91]. Representative long echo time ^1^H-MRS spectra acquired from deep frontal white matter using a multivoxel approach. The spectra are original data generated by the authors. (**a**,**b**) Spectrum obtained in an obese subject demonstrates a reduced N-acetylaspartate (NAA) to Cr ratio [NAA/Cr = 1.71] with a preserved choline (Cho) to creatine (Cr) ratio (Cho/Cr = 0.99), suggesting neuronal metabolic stress without prominent membrane turnover. (**c**,**d**) Spectrum obtained in a healthy control subject from the same region shows preserved neurometabolic balance, with normal NAA/Cr (2.90) and Cho/Cr (1.08) ratios.

An illustrative comparison of neurometabolic profiles between an obese individual and a healthy control is shown in Figure 5, highlighting reduced NAA/Cr in frontal white matter as a potential imaging-based marker of obesity-related brain vulnerability.

Myo-inositol (mI) is regarded as a glial marker and is involved in osmoregulation and intracellular signaling pathways. Elevated mI levels have been associated with astrocytic activation and neuroinflammation in several neurological disorders, including Alzheimer’s disease and demyelinating conditions [92,93,94,95,96]. Increased mI concentrations observed in obesity provide further evidence for glial involvement and sustained inflammatory processes within the brain.

At higher magnetic field strengths, MRS also allows partial assessment of glutamate and glutamine (Glx), key components of excitatory neurotransmission and cerebral energy metabolism. Disruptions in the glutamate–glutamine cycle may contribute to excitotoxicity, impaired synaptic plasticity, and cognitive dysfunction in obesity, particularly within frontal and hippocampal regions [97,98].

Taken together, MRS offers a sensitive approach for detecting early neurometabolic and neuroinflammatory brain alterations associated with obesity. By capturing biochemical changes that precede structural damage, MRS-derived biomarkers provide valuable insight into brain vulnerability and may serve as potential imaging-based markers for monitoring disease progression and therapeutic response in obesity-related brain dysfunction.

### 7.1. Single-Voxel and Multi-Voxel MRS Findings in Obesity

Both single-voxel and multivoxel proton magnetic resonance spectroscopy (^1^H-MRS) have been used to investigate neurometabolic alterations associated with obesity. Single-voxel MRS provides detailed, region-focused metabolic information, whereas multivoxel MRS enables the simultaneous assessment of neurometabolic profiles across multiple brain regions, allowing the detection of subtle, region-specific alterations that may precede overt neurological manifestations. Compared with earlier studies that predominantly relied on single-voxel approaches, multivoxel MRS offers a more comprehensive characterization of regional cerebral metabolic vulnerability in obesity [78,91,99,100].

### 7.2. Neuronal Integrity and Energy Metabolism: NAA and Creatine-Related Effects

In obesity, alterations in NAA-related metrics appear to be regionally heterogeneous. Several studies have reported preserved global NAA/Cr ratios in obese individuals, suggesting relative neuronal stability at the whole-brain level [100,101,102,103]. However, focal reductions in NAA/Cr have been observed in frontal and parietal white matter, indicating selective vulnerability of frontostriatal and executive networks [78,104,105].

Importantly, analyses based on absolute metabolite concentrations provide additional insight. While absolute NAA levels are often preserved in obesity and T2DM [106,107], discrepancies between absolute NAA and NAA/Cr ratios suggest that Cr, commonly used as a reference metabolite, may not be metabolically stable under conditions of chronic metabolic stress [91,108]. Emerging evidence indicates that increased Cr levels may reflect enhanced glial energy metabolism rather than purely neuronal activity, particularly in the context of neuroinflammation and metabolic overload [109]. This phenomenon may explain region-specific reductions in NAA/Cr without corresponding decreases in absolute NAA concentrations.

### 7.3. Choline Metabolism: Neurodegeneration Versus Neuroinflammation

In metabolic disorders, Cho alterations appear to reflect a balance between neurodegenerative and neuroinflammatory processes. Studies in insulin resistance and T2DM have reported increased Cho/Cr ratios across cortical and subcortical regions, consistent with glial activation and neuroinflammation [100,101,102,105]. 

In obesity without overt glucose metabolism disturbances, Cho findings are less consistent. However, decreased Cho/Cr ratios in frontal white matter, particularly when accompanied by parallel reductions in NAA/Cr, have been interpreted as markers of early neurodegenerative processes rather than inflammation [91].

### 7.4. Myo-Inositol as a Marker of Glial Activation

In obesity without metabolic comorbidities, mI/Cr ratios may remain preserved, suggesting that isolated obesity may not be sufficient to trigger significant astroglial activation [110]. However, elevated mI/Cr levels have been consistently reported in individuals with T2DM and metabolic syndrome, particularly in frontal and cingulate regions, supporting a role for insulin resistance and chronic inflammation in glial activation [100,101,106,111,112].

Interestingly, reductions in mI/Cr following weight loss interventions, such as intragastric balloon placement, further support the reversibility of obesity-related neuroinflammatory changes and highlight mI as a potential marker of therapeutic response [110].

Myo-inositol is commonly regarded as a glia-associated metabolite on proton MRS and plays an important role in osmoregulation and intracellular signaling pathways. Elevated cerebral mI levels are frequently interpreted as reflecting astrocytic activation and gliosis and have been consistently associated with neuroinflammatory processes across a range of neurological and metabolic conditions [94,113].

Importantly, this neuroimaging interpretation should be clearly distinguished from the systemic metabolic effects of mI observed in peripheral tissues. Reduced plasma mI concentrations have been reported in insulin-resistant states, including obesity and polycystic ovary syndrome, and mI supplementation has been shown to improve insulin sensitivity and metabolic parameters in these conditions [114,115]. These beneficial peripheral effects primarily reflect its role as an insulin-sensitizing molecule and should not be conflated with direct anti-inflammatory actions within the central nervous system.

Consequently, increased cerebral mI concentrations detected by MRS in obesity should not be interpreted as contradictory to its therapeutic metabolic effects. Instead, they likely represent region-specific glial responses to chronic metabolic and inflammatory stress within the brain. This distinction underscores the importance of contextual interpretation of mI findings, integrating neuroimaging biomarkers with systemic metabolic status.

### 7.5. Regional Brain Vulnerability in Obesity

Across MRS studies, frontal white matter, particularly deep frontal regions, emerges as a key site of metabolic vulnerability in obesity [91]. Alterations in NAA/Cr and Cho/Cr within these regions may reflect early disturbances in executive and cognitive control networks, which are known to play a critical role in appetite regulation, impulse control, and decision-making [116]. These findings align with functional and structural neuroimaging studies demonstrating frontostriatal dysfunction in individuals with elevated body mass index (BMI).

The observed regional specificity underscores the importance of multivoxel approaches, as single-voxel studies may overlook localized metabolic changes that precede global brain involvement.

### 7.6. Associations with Anthropometric and Metabolic Parameters

Beyond categorical group comparisons, MRS studies have explored associations between neurometabolite levels and anthropometric and metabolic parameters, although findings remain heterogeneous. BMI has shown modest and inconsistent correlations with neurometabolites, likely reflecting its limited ability to capture body fat distribution and metabolic phenotype [91]. In contrast, markers of central obesity, such as waist circumference, appear to demonstrate stronger associations with reduced NAA/Cr ratios in frontal white matter, suggesting that visceral adiposity may be more closely linked to cerebral metabolic vulnerability [91,117].

Notably, these observations are not uniform across studies. Gazdzinski et al. [99] reported a strong negative correlation between absolute NAA concentrations and BMI in frontal, parietal, and temporal white matter, as well as in frontal gray matter, indicating a potential association between increasing adiposity and reduced neuronal metabolic integrity. In contrast, our multivoxel MRS study [91], did not demonstrate significant correlations between absolute NAA concentrations and BMI. These discrepancies likely reflect methodological differences, including different metabolite quantification methods, regional selection, and heterogeneity of metabolic profiles within obese populations. Collectively, these findings suggest that central obesity–related measures may provide more sensitive indicators of obesity-associated brain metabolic vulnerability than BMI alone.

Biochemical components of the metabolic syndrome further modulate neuroimmune and neurometabolic vulnerability. Elevated circulating lipids may contribute to region-specific metabolic alterations through lipid-driven inflammatory signaling pathways within the CNS [32]. Conversely, higher high-density lipoprotein cholesterol (HDL-C) cholesterol levels appear to exert a protective effect on neuronal metabolism, particularly in frontal white matter, consistent with its anti-inflammatory and vasculoprotective properties [91,118]. Associations with fasting glucose are generally weak in non-diabetic populations, suggesting that prolonged metabolic dysregulation may be required to induce detectable cerebral metabolic changes [91]. 

In addition to anthropometric and lipid-related parameters, markers of insulin resistance represent a critical metabolic component influencing brain neurometabolism. Fasting insulin levels and the homeostasis model assessment of insulin resistance (HOMA-IR) have been examined in relation to MRS-derived neurometabolites, although available data remain limited and region-specific. In our multivoxel MRS study using long echo time acquisitions, associations between insulin resistance markers and neurometabolic ratios are generally weak or absent at the global level. However, focal effects have been identified, indicating selective regional vulnerability rather than uniform cerebral metabolic impairment [91]. 

Importantly, our multivoxel MRS study [91] provided detailed region-specific evidence linking insulin resistance to cerebral neurometabolic alterations. Specifically, predominantly strong negative correlations were observed between fasting insulin levels and HOMA-IR and absolute concentrations of NAA and Cho in the posterior portion of the left anterior cingulate gray matter and in deep frontal white matter. In these regions, HOMA-IR demonstrated moderate associations with Cho levels, suggesting a combination of neuronal metabolic stress and altered membrane turnover. Given the pivotal role of the anterior cingulate cortex and frontal regions in cognitive and emotional regulation [119], these findings highlight selective susceptibility of higher-order cognitive networks to insulin resistance–related metabolic stress.

These observations are partly consistent with the study by Sahin et al. [101], who reported negative correlations between insulin levels and NAA/Cr in the frontal cortex, as well as between HOMA-IR and NAA/Cr in parietal white matter. Notably, apart from the study by Sahin et al. [101], our investigation [91] remains one of the very few studies to systematically explore associations between insulin resistance markers and both absolute and ratio-based neurometabolite measures using a multivoxel MRS approach, underscoring its contribution to this emerging research field.

From a pathophysiological perspective, insulin resistance is increasingly recognized as a contributor to neuronal dysfunction through mechanisms involving mitochondrial impairment, increased oxidative stress, disrupted intracellular signaling, and DNA damage, processes that may facilitate neurodegenerative cascades [120]. Available evidence indicates that insulin resistance–associated brain metabolic changes are focal, method-dependent, and preferentially affect frontal and cingulate regions, reinforcing the importance of comprehensive metabolic profiling when assessing obesity-related brain vulnerability [91].

### 7.7. Implications for Imaging-Based Biomarkers

Collectively, MRS findings suggest that obesity-related brain alterations are subtle, region-specific, and strongly modulated by metabolic comorbidities such as insulin resistance and dyslipidemia. Rather than reflecting uniform neuronal loss, these changes likely represent a dynamic interplay between altered energy metabolism, glial activation, and early neurodegenerative processes. Multivoxel MRS-derived markers, particularly NAA/Cr, Cho/Cr, and mI/Cr in frontal and cingulate regions, hold promise as imaging-based biomarkers for identifying early brain vulnerability and monitoring metabolic and therapeutic interventions in obesity-related brain dysfunction.

An overview of MRS-derived neurometabolic alterations, their regional specificity, and associated findings from complementary neuroimaging modalities is summarized in Table 1.

### 7.8. Effects of Therapeutic Interventions on MRS and Multimodal Neuroimaging Biomarkers

Therapeutic interventions targeting obesity-related metabolic and inflammatory dysregulation may induce measurable changes in brain neurometabolism detectable by MRS, including partial normalization of spectroscopic markers associated with diabetes [110]. Lifestyle modification, caloric restriction, and weight-loss interventions have been associated with partially reversible obesity-related hypothalamic dysfunction and altered brain activity, supporting the concept that obesity-related brain vulnerability is dynamic and potentially modifiable [16].

Structural MRI evidence indicates that both overall and central obesity are associated with premature brain ageing, lower gray matter volume, and higher white matter hyperintensity burden, with these associations partially mediated by cardiometabolic and inflammatory measures, supporting a metabolically linked and potentially modifiable brain phenotype [121].

### 7.9. Limitations of Magnetic Resonance Spectroscopy in Obesity Research

Despite its unique ability to provide in vivo insight into brain neurometabolism, MRS has several important limitations that should be considered when interpreting findings in obesity research. MRS is characterized by relatively low spatial resolution and limited anatomical specificity, which may obscure regionally heterogeneous metabolic alterations. Quantification of neurometabolites can be influenced by technical factors, including magnetic field strength, voxel placement, partial volume effects, and acquisition parameters, contributing to inter-study variability. Furthermore, MRS-derived changes are indirect and require cautious biological interpretation, as alterations in metabolite levels may reflect overlapping neuronal, glial, and metabolic processes. These limitations underscore the importance of standardized acquisition protocols and the integration of MRS with complementary neuroimaging modalities to improve interpretability and translational relevance [122].

## 8. Complementary Neuroimaging Biomarkers Beyond MRS

While MRS provides direct insight into obesity-related neurometabolic alterations, increasing evidence indicates that obesity-associated brain vulnerability is best characterized through a multimodal neuroimaging framework. Complementary imaging techniques, including diffusion tensor imaging (DTI), functional magnetic resonance imaging (fMRI), volumetric MRI, cerebral perfusion imaging, and positron emission tomography (PET), capture distinct yet interconnected aspects of brain structure, function, and molecular signaling. Together, these modalities extend beyond neurometabolic changes alone and help contextualize MRS findings within broader pathophysiological processes such as impaired tissue microstructure, network-level dysfunction, brain aging, and altered neurotransmission. Recent population-based MRI study emphasizes the complementary value of integrating structural brain measures with cardiometabolic and inflammatory profiling to capture obesity-related brain vulnerability and potential premature brain ageing across biological scales [121].

### 8.1. Diffusion Tensor Imaging and Glymphatic Dysfunction

Recent DTI studies have expanded the scope of obesity-related brain research by targeting the glymphatic system, a perivascular clearance pathway essential for the removal of metabolic waste and neurotoxic proteins. Using diffusion tensor image analysis along the perivascular space (DTI-ALPS), Park et al. demonstrated that neurologically healthy individuals with obesity exhibit significantly reduced DTI-ALPS index values compared with normal-weight and overweight controls, suggesting impaired glymphatic clearance efficiency [123]. Importantly, these alterations were detected in the absence of overt neurological disease, suggesting that glymphatic dysfunction may represent an early and subclinical marker of obesity-related brain vulnerability.

The glymphatic system plays a critical role in maintaining cerebral homeostasis, particularly during sleep, and its dysfunction has been implicated in the accumulation of amyloid-β and other neurotoxic metabolites [124]. In this context, reduced glymphatic efficiency in obesity may provide a mechanistic link between chronic metabolic stress, neuroinflammation, and increased susceptibility to neurodegenerative processes [123]. These findings complement MRS-based evidence of early metabolic stress by highlighting impaired clearance mechanisms as an additional pathway contributing to obesity-related cerebral vulnerability. Consistent with this broader pattern of microstructural vulnerability, recent DTI study has demonstrated obesity-related alterations across multiple subcortical gray matter regions, including the hypothalamus, hippocampus, pallidum, and basal ganglia, supporting the concept that obesity-associated brain changes extend beyond focal pathways and reflect widespread microstructural involvement [125]. Recent diffusion-based spectrum imaging study further supports this concept by demonstrating that increased visceral and subcutaneous abdominal adiposity is associated with elevated markers of neuroinflammation and reduced axonal density across widespread white matter tracts in cognitively normal midlife individuals, highlighting a direct link between systemic adiposity and brain microstructural inflammation [126]. Recent quantitative MRI evidence further supports this concept by demonstrating early, region-specific microstructural and neuroinflammatory alterations within metabolically vulnerable regions such as the hypothalamus and hippocampus in individuals with obesity, even in the absence of overt cognitive impairment [127].

### 8.2. Volumetric MRI and Cardiometabolic Mediation

Volumetric MRI analyses provide further evidence that obesity is associated with adverse structural brain outcomes, particularly when cardiometabolic dysregulation is present. In a large population-based study, Zhou et al. reported that both general and central obesity were associated with reduced gray matter volume and increased white matter hyperintensity burden, corresponding to an estimated acceleration of brain aging by several years [121]. These findings suggest that excess adiposity may contribute to cumulative structural brain changes over time.

Crucially, mediation analyses revealed that cardiometabolic and inflammatory factors, including glycemic indices, blood pressure, triglycerides, leukocyte count, and high-density lipoprotein cholesterol, accounted for a substantial proportion of the observed associations between obesity and brain structural measures. This indicates that volumetric brain alterations are not solely driven by adiposity itself, but rather by its downstream metabolic and inflammatory consequences [121]. In the context of multimodal imaging, volumetric changes may represent relatively late manifestations of prolonged metabolic stress [121], while neurometabolic alterations detectable by MRS could emerge earlier in the disease con-tinuum [91].

### 8.3. Functional MRI and Network-Level Alterations

Functional MRI studies have provided important insights into obesity-related alterations in brain network organization, particularly within systems involved in reward processing, salience detection, and executive control. A comprehensive review by Drelich-Zbroja et al. summarized consistent abnormalities in both task-related activation and resting-state functional connectivity in individuals with obesity [128]. Task-based fMRI studies frequently demonstrate altered neural responses to food-related visual and gustatory stimuli within the orbitofrontal cortex, anterior cingulate cortex, insula, striatum, and limbic regions, reflecting dysregulated reward valuation and impaired cognitive control of eating behavior [70,128].

Resting-state fMRI findings further indicate disrupted connectivity within the default mode, salience, and executive control networks, suggesting that obesity-related functional alterations extend beyond stimulus-driven responses and reflect more generalized network reorganization [128]. Notably, many of these regions overlap with areas showing neurometabolic and structural vulnerability, supporting the notion that metabolic stress may translate into altered network dynamics [91,128]. Functional MRI thus provides a critical link between biochemical and structural brain changes and their behavioral and cognitive consequences.

### 8.4. Cerebral Perfusion Imaging and Neurovascular Dysfunction

Cerebral perfusion imaging provides complementary insight into obesity-related brain dysfunction by capturing alterations in regional cerebral blood flow (rCBF), a sensitive marker of neurovascular and metabolic coupling. Using resting-state single-photon emission computed tomography (SPECT) imaging, Silvah et al. demonstrated significant rCBF alterations in obese individuals compared with lean controls, characterized by reduced perfusion within frontoparietal regions involved in cognitive control, alongside increased rCBF within the default mode and salience networks [129].

Importantly, perfusion abnormalities were observed in regions overlapping with networks implicated in executive control, salience detection, and reward processing, including the anterior cingulate cortex, insula, prefrontal cortex, and limbic structures [129]. Associations between cerebral perfusion changes and metabolic parameters, together with evidence linking metabolic dysregulation to subclinical brain aging, further support a link between neurovascular dysfunction and systemic metabolic dysregulation [50,129]. Recent evidence further supports this association, demonstrating that higher intake of ultra-processed foods is linked to altered cerebral perfusion patterns and increased inflammatory burden, underscoring the sensitivity of perfusion imaging to diet-related neurovascular and inflammatory changes [130]. Consistent with these human findings, translational models demonstrate that diet-induced obesity accelerates age-related disturbances in cerebral perfusion, white matter microstructure, and functional connectivity, accompanied by enhanced neuroinflammatory activation and earlier cognitive decline [131].

### 8.5. Positron Emission Tomography and Molecular Targets

Positron emission tomography enables in vivo assessment of molecular pathways implicated in obesity-related brain dysfunction that are not accessible through MRI-based techniques. Using a serotonin 5-HT_6_ receptor–targeted PET tracer, Courault et al. demonstrated increased cerebral tracer binding following high-fat diet exposure, particularly within the hippocampus, striatum, cingulate cortex, temporal cortex, and brainstem [132]. These findings indicate obesity-associated modulation of central serotonergic signaling, a system closely involved in appetite regulation, reward processing, mood, and cognitive function.

Alterations in 5-HT_6_ receptor availability may reflect adaptive or maladaptive neurochemical responses to chronic metabolic stress and dietary factors. Importantly, PET imaging provides molecular-level evidence that complements MRS-detected metabolic changes and fMRI-observed functional alterations, supporting the role of neurotransmitter dysregulation in obesity-related brain dysfunction [133]. However, recent multimodal PET study indicate that obesity is associated with increased cerebral metabolic activity and resting-state brain activity without corresponding increases in translocator protein (18 kDa; TSPO) availability, suggesting that PET-based markers of neuroinflammation may have limited sensitivity in detecting obesity-related inflammatory processes and should be interpreted with caution [134]. Together, these findings highlight the value of PET as a tool for identifying specific neurotransmitter systems that may serve as therapeutic targets in obesity-related brain dysfunction.

**Figure 6:**
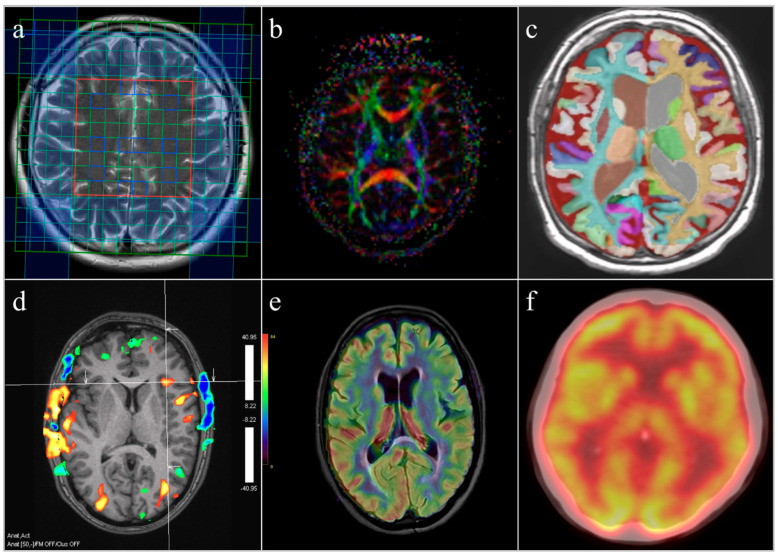
Representative examples of neuroimaging modalities used to assess obesity-related brain alterations. The figure presents representative images illustrating the principles and types of information provided by different neuroimaging techniques discussed in this review: (**a**) magnetic resonance spectroscopy (MRS) demonstrating regional neurometabolic profiles; (**b**) diffusion-based imaging illustrating microstructural tissue properties; (**c**) volumetric MRI depicting regional brain volume and cortical morphology; (**d**) functional MRI showing patterns of brain activation and functional connectivity; (**e**) cerebral perfusion imaging illustrating regional cerebral blood flow and neurovascular coupling; and (**f**) positron emission tomography (PET) demonstrating molecular and metabolic brain processes. The images are provided for illustrative purposes and do not represent original experimental data generated within a single study.

The integration of MRS-derived neurometabolic markers with structural, functional, perfusion, and molecular imaging findings underscores the value of a multimodal framework for characterizing obesity-related brain vulnerability (Figure 6).

## 9. Conclusions and Future Perspectives

Obesity is increasingly recognized as a systemic disorder with profound effects on brain structure, function, and metabolism. Accumulating evidence indicates that chronic low-grade inflammation, insulin resistance, mitochondrial dysfunction, vascular impairment, and altered brain clearance mechanisms converge to promote cerebral vulnerability in individuals with obesity. These processes preferentially affect brain regions involved in metabolic regulation, cognition, and reward processing, contributing to cognitive dysfunction and increased susceptibility to neurodegenerative disease. Integrative evidence further suggests that obesity-related memory impairment arises from converging metabolic, inflammatory, and insulin signaling disturbances affecting hippocampal and fronto-limbic circuits, reinforcing obesity as a biological contributor to cognitive vulnerability [135].

Advanced neuroimaging techniques have substantially improved our understanding of obesity-related brain alterations. MRS provides unique in vivo insight into early neurometabolic and neuroinflammatory processes that may precede overt structural damage, while complementary modalities, including DTI, volumetric MRI, fMRI, cerebral perfusion imaging, and PET, reveal microstructural, functional, hemodynamic, and molecular alterations that together define a multimodal signature of obesity-related brain vulnerability.

From a clinical perspective, the integration of MRS-derived neurometabolic markers with other neuroimaging biomarkers holds promise for identifying early and potentially reversible brain changes in obesity, supporting risk stratification and monitoring of therapeutic interventions. Perfusion and diffusion-based imaging further highlight neurovascular and microstructural dysfunction as complementary pathways linking metabolic dysregulation to cerebral vulnerability.

Overall, neuroinflammation should be regarded primarily as a downstream consequence of systemic metabolic, hormonal, vascular, and genetic disturbances associated with obesity, rather than as an isolated primary cause of brain pathology. Although neuroinflammatory changes typically arise secondary to chronic metabolic dysregulation, insulin resistance, vascular dysfunction, and impaired brain clearance mechanisms, they may subsequently amplify neuronal and glial dysfunction, contributing to a self-perpetuating cycle that increases vulnerability to cognitive decline and neurodegenerative disease.

Despite its considerable potential, MRS must be interpreted in light of important methodological and biological limitations, including limited spatial resolution, partial volume effects, inter-study variability, and the indirect nature of neurometabolic markers. These considerations underscore the need for longitudinal, multimodal neuroimaging studies that integrate MRS with structural, diffusion-based, functional, perfusion, and molecular imaging to clarify the temporal dynamics of obesity-related brain alterations and their responsiveness to therapeutic interventions. Emphasis on clinically meaningful outcomes, such as cognitive performance, metabolic improvement, cardiovascular risk reduction, and long-term neurological health, may facilitate early identification of potentially reversible brain changes and support the development of personalized prevention and intervention strategies.

## Figures and Tables

**Table 1:** Overview of MRS-derived neurometabolic alterations in obesity and their multimodal correlates.

MRS Metabolite	Typical Direction of Change in Obesity	Predominant Brain Regions	Interpretation Consistent with the Present Review	Associated Findings from Other Neuroimaging Modalities	Potential Clinical Relevance
N-acetylaspartate (NAA)	Decrease	Frontal cortical and white matter regions, particularly the anterior cingulate cortex	Neuronal metabolic stress and reduced neuronal integrity	Reduced gray matter volume, white matter microstructural damage, and disrupted executive network connectivity	Cognitive dysfunction and increased brain vulnerability
Choline-containing compounds (Cho)	Increase	Frontal white matter and subcortical regions	Increased membrane turnover associated with neuroinflammatory processes	White matter microstructural alterations, increased white matter hyperintensities, and perfusion abnormalities	Marker of neuroinflammatory activity
Myo-inositol (mI)	Increase	Frontal and cingulate regions	Glial activation and low-grade neuroinflammation	Markers of neuroinflammation, impaired glymphatic clearance, and partial reversibility after weight loss	Indicator of glial reactivity
Glutamate/Glutamine (Glx)	Variable	Frontal and limbic regions	Altered excitatory neurotransmission and metabolic imbalance	Altered functional connectivity in limbic and executive networks and neurotransmitter modulation	Cognitive and behavioral alterations
Creatine (Cr)	Relatively stable	Frontal white matter	Cellular energy buffering reference metabolite	Altered cerebral metabolic activity and diffusion-based energy metabolism changes	Limited specificity; internal reference

## Data Availability

No new data were created or analyzed in this study. Data sharing is not applicable to this article.

## Abbreviations

The following abbreviations are used in this manuscript:

IL-6	interleukin-6
IL-1β	interleukin-1 beta
TNF-α	tumor necrosis factor alpha
ROS	reactive oxygen species
CNS	central nervous system
SFA	saturated fatty acids
TLR2/4	Toll-like receptor 2/4
IKK	IκB kinase complex
IκB	inhibitor of κB
NF-κB	nuclear factor kappa B
SOCS3	suppressor of cytokine signaling 3
MRI	magnetic resonance imaging
MRS	magnetic resonance spectroscopy
PET	positron emission tomography
NAA	N-acetylaspartate
Cho	Choline-containing compounds
Cr	Creatine
mI	Myo-inositol
Glx	glutamate and glutamine
^1^H-MRS	proton magnetic resonance spectroscopy
T2DM	type 2 diabetes mellitus
BMI	Body mass index
HDL-C	high-density lipoprotein cholesterol
HOMA-IR	homeostasis model assessment of insulin resistance
DTI	diffusion tensor imaging
fMRI	functional magnetic resonance imaging
DTI-ALPS	diffusion tensor image analysis along the perivascular space
rCBF	cerebral blood flow
SPECT	single-photon emission computed tomography
HPA	hypothalamic–pituitary–adrenal
11β-HSD1	11β-hydroxysteroid dehydrogenase type 1
DHEA	dehydroepiandrosterone
DHEAS	dehydroepiandrosterone sulfate
mtDNA	mitochondrial DNA
NAD^+^	Nicotinamide adenine dinucleotide
NADPH	Nicotinamide Adenine Dinucleotide Phosphate
TSPO	translocator protein (18 kDa)
NAFLD	non-alcoholic fatty liver disease
BBB	blood–brain barrier
CHD	coronary heart disease
CV	cardiovascular
Th1	T helper 1 cells
Th17	T helper 17 cells
